# The state of health research governance in Africa: what do we know and how can we improve?

**DOI:** 10.1186/s12961-020-00676-9

**Published:** 2021-01-22

**Authors:** Juliet Nabyonga-Orem, James Avoka Asamani, Micheal Makanga

**Affiliations:** 1 Inter-Country Support Team for Eastern and Southern Africa, UHC Life Course Cluster, World Health Organization, 82-86 Enterprise/Glenara Roads, Highlands, P.O Box CY 348; Causeway, Harare, Zimbabwe; 2 The European & Developing Countries Clinical Trials Partnership (EDCTP), Anna van Saksenlaan 51, 2593 HW The Hague, The Netherlands

**Keywords:** Health research, Regulation, Research governance

## Abstract

**Background:**

The developments in global health, digital technology, and persistent health systems challenges, coupled with global commitments like attainment of universal health coverage, have elevated the role of health research in low- and middle-income countries. However, there is a need to strengthen health research governance and create a conducive environment that can promote ethics and research integrity and increase public trust in research.

**Objective:**

To assess whether the necessary structures are in place to ensure health research governance.

**Methods:**

Employing a cross-sectional survey, we collected data on research governance components from 35 Member States of the World Health Organization (WHO) African Region. Data were analysed using basic descriptive and comparative analysis.

**Results:**

Eighteen out of 35 countries had legislation to regulate the conduct of health research, while this was lacking in 12 countries. Some legislation was either grossly outdated or too limiting in scope, while some countries had multiple laws. Health research policies and strategies were in place in 16 and 15 countries, respectively, while research priority lists were available in 25 countries. Overlapping mandates of institutions responsible for health research partly explained the lack of strategic documents in some countries. The majority of countries had ethical committees performing a dual role of ethical and scientific review. Research partnership frameworks were available to varying degrees to govern both in-country and north–south research collaboration. Twenty-five countries had a focal point and unit within the ministries of health (MoH) to coordinate research.

**Conclusion:**

Governance structures must be adaptive to embrace new developments in science. Further, strong coordination is key to ensuring comprehensiveness and complementarity in both research development and generation of evidence. The majority of committees perform a dual role of ethics and scientific review, and these need to ensure representation of relevant expertise. Opportunities that accrue from collaborative research need to be seized through strong MoH leadership and clear partnership frameworks that guide negotiations.

## Background

Emerging and re-emerging infectious diseases, coupled with commitments to achieving universal health coverage and the growing interest in global health, have accentuated the role of health research in low- and middle-income countries. In the context of evolving population health needs, additional considerations include the need for contextualized evidence to generate local solutions, innovation to improve efficiencies, and the development of more effective treatment regimens given the increasing resistance to commonly used medicines. Various contextual factors call for continual adaptation of health research systems to ensure relevance and effectiveness, for example in conflict settings [1], multilayered governance in implementation research [2], and rapidly evolving digital technology. As Kostikova states, ‘…we need a new approach to science and health research’, highlighting the opportunities afforded by digitalization as the creation of real-time big data streams, social media, infectious disease modelling, and the need to embrace multidisciplinary approaches [3]. Nuyens (2005) argues that health research is a core component of improving health, equity, and development [4]. However, the suboptimal research capacity in African countries has been a long-standing concern, mainly attributable to inadequacies in funding, skills, and infrastructure, as well as weak health research governance [5–7]. Perhaps this explains the low level of research output—Africa accounts for only 2% of the world’s research output [8] and 1.3% of global publications [9].

The low domestic financing for health research is a persistent problem in Africa, despite commitments made in the past. In the Algiers Declaration of 2008, African governments committed to investing at least 2% of their health budgets in health research. However, an assessment conducted a decade later in 2018 showed that only 2 out of 39 countries that participated in the assessment within the WHO African Region (WHO/AFR) had met the target [6]. As a result, health research in Africa has been predominantly externally funded and this presents both opportunities and challenges. Opportunities are in the form of skills and technology transfer, research infrastructure development, and sharing of resources [5, 7]. Challenges, on the other hand, have been cited as failure to address priority evidence gaps, lack of local ownership [7], and exploitative research partnerships, focusing on publications as opposed to capacity-building [5]. There are documented examples of lapses and exploitive research efforts, such as failure to obtain consent and ethical approval in studies involving human subjects [10, 11], and governance issues, corruption and political instability [7], underscoring the need for strengthened research regulatory capacity in African countries. If not addressed, these challenges could substantially harm public confidence in health research, which would ultimately undermine discovery and innovation in health and health service delivery.

Several frameworks have been proposed for strengthening health research capacity; common threads in these include building capacity at the individual (training and skills) and institutional (infrastructure) levels, retention of skilled researchers, promoting partnerships in research (south–south and north–south), building networks of excellence, providing funding for research, promoting uptake of research evidence, and a favourable political environment [12–14]. Ghaffar et al. [13] noted the need for a change in the mindset of funders and international organizations to allow for funding in least considered areas such as salary top-ups and conference attendance. They further emphasized the need for a collegial and complementary approach to building collaborations within southern institutions to facilitate effective negotiations for equal partnerships with northern institutions, as opposed to competing for resources.

The WHO/AFR builds on the work of Pang et al. and proposes research capacity-strengthening interventions in four domains, namely strengthening research governance, creating and sustaining resources, producing and using health research, and financing health research [15]. Although all interventions are important, this paper focuses on health research governance, which we consider a prerequisite to building sustainable research capacity. As Stiglitz observed, ‘Where good stewardship exists, health research and its utilization have apparently flourished’ [16]. This assertion is supported by Maïga et al., who noted that ‘without independent strong regulatory and ethical oversight of clinical trials, the safety of research subjects and scientific integrity of clinical data cannot be verified’ [17]. The African Union further emphasizes the importance of stakeholder coordination and strong governance structures to promote ethics and research integrity and to increase public trust in research [18].

Health research governance encompasses a number of parameters that should provide a conducive environment for the coordination and conduct of health research. Several frameworks propose interventions to be pursued in strengthening research governance, and common among these is articulation of a vision for health research, setting research priorities, coordination of actors’ interventions and investments, ensuring ethical standards, and building research partnerships [13, 14, 19]. The WHO/AFR research strategy [15] builds on these frameworks and outlines the major components of research governance as availability of valid health research policies, strategic plans, and priority lists, legislation on health research, national or institutional ethics review committees, scientific review committees, and strong institutional capacity to coordinate research (availability of a focal person and a designated unit to coordinate health research). In addition, we underscore the importance of having collaborative agreements in research partnerships to ensure mutual benefit. The WHO/AFR research strategy underpins the analysis presented in this paper, given that this assessment was undertaken to monitor the progress of the implementation of the WHO/AFR research strategy adopted by the ministers of health in the Member States of the WHO/AFR in 2014.

The objective of this paper is to assess whether the required structures as outlined in the WHO/AFRO research strategy are in place in the various countries to ensure a conducive environment for conducting health research. We focus on the presence of the governance structures, or lack thereof, as opposed to their implementation or functionality. As much as the latter is important, we believe having these in place lays the foundation.

## Methods

We employed a cross-sectional survey design using a semi-structured mailed questionnaire.

### Study setting

The study was undertaken in the Member States of the WHO/AFR. These are the countries that had endorsed and committed to implementing the WHO/AFR research strategy and tasked the WHO Regional Office for Africa (WHO/AFRO) with regular monitoring of the commitments therein.

### Data collection

The survey questionnaire was emailed to all 47 Member States of WHO/AFR, and 35 responded (response rate of 74%). Prior to undertaking the survey, country teams (ministry of health [MoH] research focal person/designee, head of the national health research institute, and a researcher from one of the research institutes who was selected by the MoH) were oriented on the objectives of the assessment and the commitments detailed in the WHO/AFRO research strategy which were to be monitored on a regular basis (these informed the survey instruments), and were trained on completing the questionnaire. The questionnaire was emailed to the WHO Country Office focal person in charge of health research, who then shared it for completion with the person who was in charge of coordinating health research in the country, which was either the MoH focal point for research in the country or the head of the national health research coordination institute, as the arrangements differed from one country to another. The completed questionnaire was validated by an in-country team comprising representatives of institutions conducting health research, the head of the national research coordination institution, the focal point for research in the WHO Country Office, and the focal point for research in the MoH. The WHO Country Office focal point for research emailed the completed questionnaire to the WHO/AFRO.

Data were collected on the different parameters that foster health research governance, as shown in Table 1. Data were collected between December 2017 and August 2018.

**Table 1:** Details of data collected

		Parameter assessed
1.	Countries with legislation on research for health	Presence of laws and legislation regulating the conduct of health research, whether as stand-alone or embedded in other laws, whether the laws included considerations for ethics in research, date enacted, number of laws/legislation in place
2.	Health research policy and health research strategic plan	Availability of a health research policy and strategic plan, validity period, whether disseminated, and whether under implementation. If still under development, the current status
3.	Research priority list	Availability, validity period
4.	National and institutional ethics review committees and scientific review committees	Availability of ethics and scientific review committees at the national, institutional, and service delivery (hospital) levels. (The role of ethical committees is to ensure protection of potential participants in the research and consider the potential risks and benefits for the community in which the research will be carried out. The role of the scientific committee is to ensure scientific rigour of proposals/protocols). In the majority of cases, the committees perform a dual role
5.	Institutional capacity to coordinate health research	Availability of a health research focal person in the country (either based in the ministry of health or a national health research institute) and health research promotion unit/directorate within the ministry of health
6.	Availability of frameworks to foster effective partnerships	These were assessed at two levels: 1. Partnership frameworks/guidelines/agreements to guide collaborations between national research institutions and foreign (outside the country) institutions and agencies 2. At the national level—availability of memoranda of understanding between ministries of health and universities and national research institutes

### Data cleaning and analysis

Data were cleaned by the team put together by the WHO/AFRO who were in charge of data analysis. Data were analysed using basic descriptive and comparative analysis.

## Results

### Availability of research laws and legislation

As shown in Table 2, 18 out of 35 (51.4%) countries had existing laws or legally binding regulations in place to regulate health research, albeit in varied forms. In some of the countries (Gambia and the Congo), regulatory aspects were embedded in policy documents which were not legally binding, while in some countries several laws were established—there were two laws each in Cameroon, Ghana, Gabon, and Kenya, while Rwanda had five laws. Twelve out of 35 countries (34.3%) had no specific laws to regulate the conduct of health research. In five of the countries (14.3%), the development of such laws was still ongoing at the time of the survey, which in some cases was a protracted process. In Lesotho, for instance, the process of enacting a comprehensive public health act that would incorporate issues on health research had been ongoing for 2 years and was yet to be completed.

**Table 2:** Availability of laws and regulations regulating health research (*N* = 35 countries)

Have research laws and legislation	Laws and legislation in draft form	Lack of research laws and legislation
Kenya, Gambia, Malawi, Mali, Mauritius, Rwanda, United Republic of Tanzania, Uganda, Zambia, Zimbabwe, Nigeria, Cameroon, Gabon, Congo, Benin, Senegal, Ghana, Mozambique (18)	Lesotho, Liberia, Sierra Leone, Swaziland, Niger (5)	Ethiopia, Botswana, Namibia, Seychelles, South Sudan, Guinea-Bissau, Côte d’Ivoire, Burundi, Mauritania, Democratic Republic of the Congo, Cape Verde, Eritrea (12)

The legal regimes for health research varied considerably. For example, in Rwanda, the mandate to enforce the different laws lay with different line ministries and government institutions. In some countries the laws were narrow in scope—restricted to regulating clinical trials (Mauritius), biomedical research on humans (Mali), and ethics of research (Senegal). In countries where legislation was in place, this was enacted between 1979 (such as the Parliamentary Act No. 23 establishing the National Institute for Medical Research in the United Republic of Tanzania) and 2017.

### Availability of health research policy, research strategic plan, and research priority lists

Sixteen countries (~ 46%) had research policies that were under implementation (see Table 3). In four of the countries (Liberia, Namibia, South Sudan, Nigeria), although policies were developed, they had never been disseminated. In the case of Gambia, the development process was protracted (over 5 years) and the policy was categorized as ‘expired’ while still in draft form. In the United Republic of Tanzania, although the policy was expired, it had never been implemented, as stated, ‘*National Research Policy developed under the Ministry of Science and Technology in 2010, has not been updated to date and was rarely referred to*’, and the cited shortcoming was the perceived insufficiency in some aspects—‘*was not sufficient to include technological development*’. In Niger, the policy was broad, encompassing both health and education. In Ethiopia there was reportedly no policy, which was attributed to overlapping mandates between the MoH and Ministry of Science and Technology, and the lack of a coordination unit at the MoH.

**Table 3:** Availability of strategic documents to guide health research (*n* = 35 countries)

Availability of strategic document	Status				
	Available, disseminated and under implementation	Available but not disseminated and not implemented	Outdated/expired	Draft	None
Health research policy	Benin, Cameroon, Congo, Côte d’Ivoire, Lesotho, Mali, Rwanda, Swaziland, Senegal, Ghana, Uganda, Zambia, Zimbabwe, Niger, Mozambique, Eritrea (16)	Liberia, Namibia, South Sudan, Nigeria (4)	Gambia, United Republic of Tanzania (2)	Kenya, Malawi, Gabon (3)	Burundi, Cape Verde, Ethiopia, Botswana, Democratic Republic of the Congo, Mauritius, Mauritania, Sierra Leone, Seychelles, Guinea-Bissau (10)
Health research strategic plan	Kenya, Lesotho, Malawi, Mali, Rwanda, Seychelles, Swaziland, United Republic of Tanzania, Ghana, Niger, Cameroon, Benin, Senegal, Mozambique, Eritrea (15)	Liberia, Côte d’Ivoire (2)	Gambia, Uganda, Zambia, Burundi (4)		Ethiopia, Botswana, Mauritius, Namibia, Sierra Leone, South Sudan, Zimbabwe, Nigeria, Guinea-Bissau, Democratic Republic of the Congo, Gabon, Congo, Mauritania, Cape Verde (14)
Research priority list	Ethiopia, Botswana, Lesotho, Liberia, Malawi, Rwanda, South Sudan, Swaziland, United Republic of Tanzania, Zambia, Zimbabwe, Nigeria, Ghana, Cameroon, Congo, Côte d’Ivoire, Benin, Burundi, Mauritania, Senegal, Democratic Republic of the Congo, Niger, Cape Verde, Mozambique, Eritrea (25)		Kenya, Uganda, Guinea-Bissau (3)		Gambia, Mali, Mauritius, Namibia, Sierra Leone, Seychelles, Gabon (7)

In 15 countries (~ 43%) there were strategic plans for health research which were at various stages of implementation, but these were lacking in 14 countries (40%). However, those that were available existed in several forms, with some embedded in health sector strategic plans (Kenya, Seychelles, and United Republic of Tanzania) and others embedded in action plans and strategies of other line ministries (Ministry of Higher Education and Scientific Research in the case of Burundi). In some countries such as Gambia, the research strategic plan was considered ‘expired on arrival’, as its horizon had lapsed while still in draft form, and hence it was never implemented. In contrast, the research strategic plans for other countries like Uganda and Zambia were outdated but still used in guiding investments and interventions on health research. In Ethiopia, they relied on the strategic plan of the health research institute to provide strategic direction for health research.

The majority of countries (25/35) had research priority lists, although these varied in comprehensiveness. For example, the research priority list in Ethiopia only addressed the research needs of some programs as opposed to sector priorities.

Eight of the countries (~ 23%) had neither health research policy nor health research strategic plan but had a research priority list (or national research agenda). In three of the countries (Mauritius, Sierra Leone, and Gabon) there was no national health research policy, strategy, or priority list.

### Availability of ethical and scientific committees

Availability of ethical committees is a critical component in ensuring ethical standards and protection of human subjects, while scientific committees ensure rigour and integrity of research. The availability of such committees in the assessed countries is shown in Table 4

**Table 4:** Availability of ethical and scientific committees

Availability of scientific and ethical committees	Availability of scientific and ethical committees only at the national level	Availability of scientific and ethical committees at institutions and service delivery levels
Benin, Burundi, Botswana, Cape Verde, Cameroon, Congo, Democratic Republic of the Congo, Eritrea, Ethiopia, Eswatini, Gabon, Gambia, Ghana, Guinea-Bissau, Kenya, Lesotho, Liberia, Malawi, Mali, Mauritania, Mauritius, Mozambique, Namibia, Niger, Nigeria, Gambia, Rwanda, Sierra Leone, Senegal, Seychelles, South Sudan, United Republic of Tanzania, Uganda, Zambia, Zimbabwe (35)	Gambia, Mauritius, Sierra Leone, South Sudan, Guinea-Bissau, Gabon, Burundi, Mauritania, Niger, Cape Verde (11)	Benin, Botswana, Cameroon, Congo, Democratic Republic of the Congo, Kenya, Ethiopia, Ghana, Eritrea, Lesotho, Liberia, Malawi, Mali, Mozambique, Namibia, Rwanda, Seychelles, Eswatini, United Republic of Tanzania, Uganda, Zambia, Zimbabwe, Nigeria, Senegal (24)

All countries had scientific and ethical committees; 11 were at the national level only, while 24 countries had these at the institution and service delivery level as well.

### Availability of frameworks to foster effective partnerships

The WHO/AFR research strategy advises countries to forge research partnerships at two levels: (1) with relevant international research institutions, collaborators, and funding agencies, and (2) at the country level between the MoH, national health research institutions, and training institutions. As a proxy for existing partnerships, the assessment focused on the availability of collaborative frameworks/guidelines for engagement with international actors and memoranda of understanding (MoU) to guide engagements with in-country actors.

As shown in Table 5, in 15 out of the 35 countries (~ 43%), there was a collaborative engagement framework to guide partnerships with foreign agencies/institutions in undertaking research. Fourteen and 20 countries had MoUs with the national research institute and universities, respectively.

**Table 5:** Availability of partnership frameworks

Have collaboration frameworks/guidelines to guide engagements with agencies outside the country	Have a memorandum of understanding between ministry of health and the national research institute	Have a memorandum of understanding between ministry of health and university institutes	Lack of MoU with a health research institute and lack of collaborative agreements
Kenya, Botswana, Mali, Rwanda, South Sudan, Eswatini, Ghana, Cameroon, Gabon, Congo, Benin, Senegal, Democratic Republic of the Congo, Niger, Mozambique (15 countries)	Liberia, Malawi, Mali, Rwanda, Mauritius, Uganda, Zambia, Ghana, Cameroon, Gabon, Senegal, Niger, Cape Verde, Mozambique (14 countries)	Kenya, Gambia, Liberia, Mali, Mauritius, Rwanda, Eswatini, United Republic of Tanzania, Uganda, Zimbabwe, Ghana, Cameroon, Guinea-Bissau, Gabon, Congo, Mauritania, Senegal, Niger, Cape Verde, Mozambique (20 countries)	Namibia, Nigeria, Ghana, Côte d’Ivoire, Burundi, Mauritania, Eritrea (7 countries)

However, seven countries (20%) lacked both collaborative agreements and MoUs to guide engagements with national research institutions. In other countries such as Zambia, the development of collaborative agreements was ongoing at the time of the assessment, while in the United Republic of Tanzania, collaboration with foreign agencies was said to be negotiated at the level of the local partner institution and detailed in MoUs.

The lack of an MoU between the MoH and the national health research institute, however, needs to be interpreted with caution. The majority of countries without this in place made reference to an understanding that this was not necessary given that both entities were government institutions, and working relationships were not constrained in any way. Some countries have no national research institutions (Lesotho, Namibia, Seychelles, Sierra Leone, Eswatini, Eritrea). In the case of Gambia, the ownership of the research institute was reportedly undefined, making it difficult to forge a partnership with the MoH.

Across countries where they were available, collaborative agreements with international agencies/institutions mainly addressed the issue of data sharing, while the MoUs between the MoH and universities (which were mostly with public universities) addressed areas of human resource development, providing technical advice, and undertaking research. MoUs with research institutions addressed undertaking research on behalf of the MoH and providing technical assistance.

### Institutional capacity to coordinate health research

This dimension of the assessment focused on the availability of a research department within the MoH with a designated focal point and having a program of work to champion the health research agenda of the country. As shown in Table 6, 25 out of 35 countries (71.4%) had established research departments at the MOH and with designated focal points for coordinating health research in the country. Seven countries (~ 20%) only had designated research focal points, without full-fledged departments within the MoH, hence with no dedicated work plans with budgets for health research. Only two countries (Mauritius and Gabon) lacked both a focal point and a department.

**Table 6:** Availability of structures to coordinate research

Designated focal point and research department	Lack of a designated focal point and research department	Designated focal person but no department	Coordinating unit undertaking research
Senegal, Niger, Mozambique, Kenya, Botswana, The Gambia, Lesotho, Liberia, Malawi, Mali, Rwanda, Sierra Leone, South Sudan, Eswatini, United Republic of Tanzania, Cameroon, Uganda, Zambia, Zimbabwe, Ghana, Guinea-Bissau, Congo, Côte d’Ivoire, Burundi, Nigeria (25 countries)	Mauritius, Gabon (2 countries)	Democratic Republic of the Congo, Cape Verde, Eritrea, Namibia, Seychelles, Benin, Mauritania (7 countries)	Senegal, Niger, Mozambique, Kenya, Liberia, Malawi, Ghana, Cameroon (8 countries)

Different arrangements were also reported in Cape Verde, Ethiopia, and Kenya, where the coordination of health research was assigned to the national research institutes, which also had mandates to undertake health research, thereby raising potential conflict of interest. In the case of Liberia, Malawi, and Rwanda, although the research coordination units were within the MoH, they were also undertaking research, mainly implementation research. Across nearly all countries, research departments were noted to be underfunded, which undermined their capacity to coordinate and/or supervise health research.

## Discussion

### Availability of research laws and legislation

The assessment revealed that only slightly more than half of the countries had legislation to regulate the conduct of health research, with as many as 12 countries having no specific legislation. In other countries, the legislative void was filled by embedding health research regulatory frameworks within nonbinding health policies. The lack of legislation to regulate health research has been previously noted to hamper multi-institutional research [20]. Even for countries with legislation in place, some were either grossly outdated—such as a four-decades-old law in the United Republic of Tanzania—or too limiting in scope, like the single focus on clinical trials in Mauritius. Hence, these can be argued to be barely relevant to the challenges and opportunities in modern health research. Whitworth et al. cite the nonprogressive and restrictive legislative architecture as an important impediment to advancing health research in Africa. In their review of the research environment in sub-Saharan African countries, it was noted that legislative frameworks had not kept pace with advances like genetics research and intellectual property rights [20].

Although the current assessment did not analyse the content of the legislation that was in place in order to identify the specific gaps, it is not unreasonable to assume that narrowly focused health research legislation or outdated laws are likely to have inherent gaps for the levels of sophistication and methodological pluralism employed in modern health research. Advancements in digital technology have been witnessed in the recent past as a means to improve access to quality services and as an opportunity to advance health research. Outdated or restrictive legislation hampers the government’s capacity to steward such advances, for example to ensure data protection and to leverage data and technology to advance research. As Cory and Stevens [21] note, ‘*an overly restrictive data governance framework will limit the potential of digital health technologies*’. In our assessment, the research health policy of the United Republic of Tanzania was perceived as inadequate, with a negative impact on implementation, as respondents noted that ‘*the policy was not sufficient to include technological development*’.

Also, a number of countries reported having dual or multiple legislation on health research, which may also pose a risk of legislative conflict, especially when institutional mandates derived from such legislation are not very clear or overlap. For instance, in the case of Ethiopia, the health research mandate of the MoH reportedly overlapped with that of the Ministry of Science and Technology which also had a mandate for all science-related research in the country—a situation that partly explains the lack of a clear policy direction for health research. Chocarro and Folb [22] contend that lack of a clear mandate poses a great obstacle to enforcing legislation.

Another dimension is the extent to which legislation such as that for health research is enforced or complied with by actors in the health research ecosystem. Although the current study did not assess the level of compliance with the stated national legislation on health research, previous work by Yakubu and Adebamowo [23] noted, for example, that the ineffective regulation of health research in Nigeria, despite the availability of a regulatory document, had adverse implications for research and development in the country. Umeokafor et al. [24] cited political influence, bribery, and corruption among the barriers to enforcing legislation [9].

### Availability of health research policy, strategic research plan, and research priority lists

Another dimension of health research governance assessed in this study is the need for a clear and unambiguous strategic direction for health research development to be detailed in policies, strategies, and research priority lists [15]. There are countries without these in place, and where such a vacuum exists, poorly harmonized, uncoordinated, and duplication of health research activity have been reported to hamper sustainable progress in building research capacity in Africa [20]. El Achi et al. attributed the fragmentation in the health research landscape in the Middle East and North Africa Region in part to the lack of national policies and strategic plans to guide investments [25]. Kirigia et al. [26] made the same observation in reference to the national health research systems in Malawi, stating that the lack of a health research policy, strategic plan, and research for health management forum negatively impacted the effectiveness of the government’s stewardship of the research agenda.

The present assessment revealed that out of 35 countries that responded to the survey, 25 (71%), 16 (46%), and 15 (43%) had a research priority list, health research policy, and strategic plan, respectively. We emphasize, however, that in some of the countries, health research was embedded in the overall policy/strategic plan, and the extent to which this serves to effectively guide health research development cannot be ascertained. Aidam and Sombié highlighted suboptimal consultation to ensure adequate consideration for health research in such approaches [27]. A study of the 14 Economic Community of West African States (ECOWAS) countries found a more favourable picture, with 57% of countries having a policy or strategic document to guide health research development [28].

Unfortunately, we found that 15, 18, and 10 countries had no substantive national policy, strategy, or research priority, respectively, at the time of the assessment. Thus, at least one-third of the countries had no national policy or strategic direction driving the conduct, dissemination, and use of health research evidence. This situation undoubtedly not only weakens research interest in-country, but also leads to misalignment between research initiatives and the national health sector development priorities. Corroborating this assertion, respondents also reported that there were just a few nationally and institutionally initiated and funded health research studies, which they attribute in part to the absence of a well-coordinated institutional health agenda-setting mechanism. This was compounded by the fact that in at least 10 of the countries that participated in the assessment, there were no departments with the requisite staff and budget within the MoHs that were specifically tasked with coordinating the health research agenda. Even worse, in two countries no one had official responsibility for coordinating health at the MoH.

In some of the countries, research priorities were determined at a program on institutional level. While one may argue that this is perhaps better than nothing, the utility of such limited and fragmented approaches is suboptimal, as it falls short of comprehensively addressing health sector evidence gaps. Indeed, comprehensive approaches focusing on sector-wide health research priority-setting have been advocated by several scholars [29–31]. Further, the importance a nationally owned research agenda, as advocated in the Paris Declaration and Accra Agenda for Action, has been underscored, and in achieving this, we again turn to strong research governance and leadership by national governments.

### Availability of ethical and scientific committees

In line with the Nuremberg Code and Declaration of Helsinki on health research ethics, the ethical and moral conduct of researchers and the soundness of methods and safeguarding of public/participant interests is vested in the role of ethics and scientific committees [32]. All countries had research ethics and/or scientific committees that examined the scientific rigour and compliance with ethical principles of research studies. Some opportunities provided in the past may explain this favourable finding, such as the European and Developing Countries Clinical Trials Partnership (EDCTP), which has provided specific funding to develop regulatory and ethical review capacity within countries in sub-Saharan Africa, supporting activities in 27 countries.

In the majority of countries (80%), the ethical committees double as the scientific committees. There is no consensus as to whether this is the right or wrong approach, as some have argued that it can delay clearance of research protocols and increase costs [33], while others argue that what must be ensured is inclusion of relevant scientific expertise. However, Kass et al., in their review of research ethics committees (REC) in Africa, highlight the difficulty in ensuring adequate review of both the science and ethics aspects by such committees [34].

Although the region still hosts relatively few trials relative to its disease burden, more clinical research is being carried out in sub-Saharan Africa. The increasing global interest in conducting trials is placing growing pressure on poorly funded regulatory and ethical review bodies. Furthermore, increasingly complex clinical trial designs and ethical issues, such as those surrounding research on vulnerable populations, pose major challenges to national regulatory authorities as well as national and institutional RECs. Such capacity can however be fostered at a subregional level, where expertise may be pooled to serve several countries, such as the network of national ethics committees in ECOWAS countries [35].

### Availability of frameworks to foster effective partnerships

Our results reveal varied efforts at forging research partnerships both at the country level and with agencies outside the country. Out of the 35 countries that participated in the assessment, 15 (43%), 20 (57%), and 14 (40%) had agreements to promote partnerships with foreign universities and agencies, MoUs with universities, and MoUs with national research institutes, respectively. Although the availability of such agreements is a good start, realizing effective partnerships will take more than this. Indeed, a study of 15 ECOWAS countries reported that collaborations between research institutions remained ineffective despite deliberate efforts to improve collaboration between participating institutions through a research project by the West African Health Organization (WAHO) over a 5-year period (2009–2013).

The lack of such agreements in some of the countries represents a missed opportunity for effectively harnessing research resources, for example in the 20 countries that lacked collaborative agreements to guide north–south partnerships. These gaps might explain the perception of ‘exploitative research’ in north–south partnerships. Bourn et al. refer to the importance of clarity in expectations and goals if partnerships are to be equitable, and these can be detailed in collaborative agreements [36]. The global code of conduct for health research in resource-poor settings draws our attention to the fact that partnerships are indeed advantageous to both parties [20]. In addition, Chu et al. emphasize the need for meaningful partnerships with local institutions that result in mutual benefit as opposed to ‘extractive’ research [5]. The partnership of mutual benefit can only happen when partnership frameworks are devised and are implemented. Whitworth et al. also argue that such partnerships must be long-term and sustainable [20]. Mayhew et al. [37] further attest to this and emphasize the importance of personal relationships and trust between members of the partner institutions. On the other hand, partnerships between in-country universities have shown positive lessons in Nigeria [38], and should be encouraged for better synergy in in-country research efforts. Additionally, various regional efforts have proven beneficial, such as the West African Health Research Network (WAHRNET) [27] that brings together 30 research institutions and 22 medical schools in 15 ECOWAS countries and has leveraged political clout from the ECOWAS Assembly of Health Ministers. A subregional approach may offer an alternative where country-level negotiation capacity is weak. Aidam and Sombié, however, caution that such regional approaches must be coupled with adequate funding [27]. A similar approach is being championed by the African Union to harmonize medical products regulation in Regional Economic Communities blocs [39].

### Institutional capacity to coordinate health research

Twenty-five out of 35 countries (71%) had established research departments within the MoH, while eight had only focal points without departments, and two lacked both a department and a focal point. This number is fairly high in comparison to what Sombié et al. [28] found in their study of 14 ECOWAS countries in 2011, where only 50% had research directorates within the MoH. Fourteen out of 16 West African countries responded to our survey and had either a research directorate (11) or a research focal point within the MoH (3). WAHO’s efforts to improve the health research environment in the ECOWAS regions may explain this favourable finding [40]. We do however acknowledge that the presence of directorates and focal points does not imply functionality; indeed, Sombié et al. reported that among the 14 ECOWAS countries, only 7% of the directors of research units had the required training in managing research [28]. Noteworthy however is the fact that in some countries the coordinating units were also undertaking research, raising potential conflict of interest. Strong MoH institutional capacity has proven beneficial, for example in Rwanda, where inclusion of local authors is a requiremnet when using local data for all published studies [41]. Palmer et al. [42] raise another possibility that the absence or even weak MoH leadership can be attributed to the uncertainty regarding the political implications of health research coordination.

### Study limitations

Regulation of health research takes more than just the availability of laws, regulations, strategies, and structures. Our assessment focused on the existence of systems and processes rather than their content, comprehensiveness, and functionality. We argue, however, that availability is a prerequisite to functionality, thus the relevance of our study. Beyond open-ended questions in the assessment tool, no follow-up qualitative information was collected through in-depth interviews to provide a comprehensive explanation and insights into some of the issues identified. This notwithstanding, the findings from this assessment do provide clear actions to be undertaken by governments in strengthening research governance.

## Conclusion

Building strong health research governance cannot become a reality in Africa without having the prerequisites in place. This study has highlighted the key gaps across countries in the WHO/AFR which should serve as a clarion call to governments and partners. Current drawbacks that must be addressed include restricted research priority lists (only addressing program research needs) and restrictive and outdated research legislation and policies, which may be explained by gaps in consultation during the development stage. Strong coordination and stakeholder consultation are key to ensuring comprehensiveness and complementarity in both research development and generation of evidence that can adequately provide answers to policy questions.

An adaptive governance structure to embrace new developments in medicine is another consideration to guard against weakness in regulating research on new interventions. In this regard, the outdated laws can be limiting. It is thus imperative that outdated or narrowly scoped laws be updated from time to time to respond to both the challenges and opportunities presented by technological and methodological advancements impacting on or driven by health research.

Protecting research participants and ensuring scientific rigour are paramount. In this aspect, almost all countries had scientific and ethical committees in place, albeit performing a dual role in the majority of cases. Emphasis should be on ensuring representation of relevant expertise and functionality.

There is a need to seize opportunities that accrue from collaborative research through strong MoH leadership and clear partnership frameworks that guide negotiations. Partnership frameworks to guide north–south partnerships and MoHs with research institutes and universities need to be developed and enforced. Given the limited research capacity, resources available through north–south partnerships, as well as those from in-country research institutes and universities, offer opportunities that need to be embraced, learning from a similar approach in ECOWAS countries that was beneficial. A clear mandate for coordination of research must be ensured between the different government ministries and institutions.

## Data Availability

The data set generated and analysed during the current study is available from the WHO Regional Office for Africa.

## Abbreviations

MoH: Ministry of Health
MoU: Memorandum of understanding
RECs: Research ethics committees
WHO: World Health Organization
WHO/AFR: World Health Organisation, African Region
ECOWAS: Economic Community of West African Countries
WAHO: West Africa Health Organisation
